# Identification of ultra-rare disruptive variants in voltage-gated calcium channel-encoding genes in Japanese samples of schizophrenia and autism spectrum disorder

**DOI:** 10.1038/s41398-022-01851-y

**Published:** 2022-02-26

**Authors:** Chenyao Wang, Shin-ichiro Horigane, Minoru Wakamori, Shuhei Ueda, Takeshi Kawabata, Hajime Fujii, Itaru Kushima, Hiroki Kimura, Kanako Ishizuka, Yukako Nakamura, Yoshimi Iwayama, Masashi Ikeda, Nakao Iwata, Takashi Okada, Branko Aleksic, Daisuke Mori, Takashi Yoshida, Haruhiko Bito, Takeo Yoshikawa, Sayaka Takemoto-Kimura, Norio Ozaki

**Affiliations:** 1 Department of Psychiatry, Nagoya University Graduate School of Medicine, Nagoya, Japan; 2 Department of Neuroscience I, Research Institute of Environmental Medicine, Nagoya University, Nagoya, Japan; 3 Molecular/Cellular Neuroscience, Nagoya University Graduate School of Medicine, Nagoya, Japan; 4 Department of Oral Biology, Graduate School of Dentistry, Tohoku University, Sendai, Japan; 5 Protein Research Foundation, Osaka, Japan; 6 Graduate School of Frontier Biosciences, Osaka University, Osaka, Japan; 7 Department of Neurochemistry, Graduate School of Medicine, The University of Tokyo, Tokyo, Japan; 8 Laboratory for Molecular Psychiatry, RIKEN Brain Science Institute, Wako, Saitama Japan; 9 Department of Psychiatry, Fujita Health University School of Medicine, Toyoake, Japan

**Keywords:** Schizophrenia, Molecular neuroscience

## Abstract

Several large-scale whole-exome sequencing studies in patients with schizophrenia (SCZ) and autism spectrum disorder (ASD) have identified rare variants with modest or strong effect size as genetic risk factors. Dysregulation of cellular calcium homeostasis might be involved in SCZ/ASD pathogenesis, and genes encoding L-type voltage-gated calcium channel (VGCC) subunits Ca_v_1.1 (*CACNA1S*), Ca_v_1.2 (*CACNA1C*), Ca_v_1.3 (*CACNA1D*), and T-type VGCC subunit Ca_v_3.3 (*CACNA1I*) recently were identified as risk loci for psychiatric disorders. We performed a screening study, using the Ion Torrent Personal Genome Machine (PGM), of exon regions of these four candidate genes (*CACNA1C, CACNA1D, CACNA1S, CACNA1I*) in 370 Japanese patients with SCZ and 192 with ASD. Variant filtering was applied to identify biologically relevant mutations that were not registered in the dbSNP database or that have a minor allele frequency of less than 1% in East-Asian samples from databases; and are potentially disruptive, including nonsense, frameshift, canonical splicing site single nucleotide variants (SNVs), and non-synonymous SNVs predicted as damaging by five different *in silico* analyses. Each of these filtered mutations were confirmed by Sanger sequencing. If parental samples were available, segregation analysis was employed for measuring the inheritance pattern. Using our filter, we discovered one nonsense SNV (p.C1451* in *CACNA1D*), one *de novo* SNV (p.A36V in *CACNA1C*), one rare short deletion (p.E1675del in *CACNA1D*), and 14 NSstrict SNVs (non-synonymous SNV predicted as damaging by all of five *in silico* analyses). Neither p.A36V in *CACNA1C* nor p.C1451* in *CACNA1D* were found in 1871 SCZ cases, 380 ASD cases, or 1916 healthy controls in the independent sample set, suggesting that these SNVs might be ultra-rare SNVs in the Japanese population. The neuronal splicing isoform of Ca_v_1.2 with the p.A36V mutation, discovered in the present study, showed reduced Ca^2+^-dependent inhibition, resulting in excessive Ca^2+^ entry through the mutant channel. These results suggested that this *de novo* SNV in *CACNA1C* might predispose to SCZ by affecting Ca^2+^ homeostasis. Thus, our analysis successfully identified several ultra-rare and potentially disruptive gene variants, lending partial support to the hypothesis that VGCC-encoding genes may contribute to the risk of SCZ/ASD.

## Introduction

Schizophrenia (SCZ) is a severe, chronic, and common psychiatric disorder that is characterized by psychotic symptoms such as hallucinations and delusions; the prevalence of SCZ is estimated to be 1% [1]. Autism spectrum disorders (ASDs) are a range of conditions characterized by repetitive patterns of behavior and interests, as well as persistent deficits in social communication and interaction [2]. SCZ/ASD both have been implicated to have a high heritability that is estimated as 60–90% from population-based and twin studies [3, 4]. Recently, genomic studies coupled with large-scale collaborative projects have identified hundreds of common and rare mutations that contribute to SCZ/ASD [5–8].

Voltage-gated calcium channels (VGCCs) are transmembrane proteins that are activated by depolarization, triggering Ca^2+^ influx into neurons and other excitable cells [9]. VGCCs are composed of a pore-forming α_1_ subunit and auxiliary α_2_δ and β subunits⊡ The α_1_ subunit is encoded by *CACNA1* genes, including *CACNA1C* and *CACNA1D*; both of these genes have been associated with SCZ/ASD, as elucidated by several previous genetic and biological studies [5, 9–12]. Proper VGCC activity underlies many essential physiological functions, governing neuronal activity, sensory functions, and muscle contraction [9]. Notably, *CACNA1C* and *CANCA1D* are two genes that are expressed predominantly in the nervous system; the encoded Ca^2+^-permeating α_1_ subunits (Ca_v_1.2 and Ca_v_1.3, respectively) are components of channels that are located at postsynaptic somatodendritic sites [13]. Several mouse studies have provided important insights into the roles of these proteins in the pathophysiology of psychiatric disease. For example, decreased expression of Ca_v_1.2 in the forebrain has been shown to cause anxiety-like behavior in mice, suggesting that changes in expression or function of this protein may contribute to neuropsychiatric anxiety [12]. Timothy Syndrome is a rare multi-organ disease caused by a *de novo* missense mutation (p.G406R) in *CANCA1C*, and surviving patients may develop syndromic autism [14, 15]. Knock-in mice expressing p.G406R *CANCA1C* show behavioral traits reminiscent of autistic symptoms in the social domain as well as in the repetitive/restricted behavior domain [16–18].

Recent genetic studies utilizing next-generation sequencing implicated common and rare genetic variations in VGCC-encoding genes in the etiology of psychiatric disorders such as SCZ/ASD [5, 19–26]. For instance, several large-scale genome-wide association studies (GWASs) identified *CACNA1C* as a critical candidate susceptibility gene in multiple psychiatric disorders, including SCZ, autism, bipolar disorder (BD), and major depression [11]. Whole-exome sequencing studies (WESs) identified recurrent *de novo* mutations in *CACNA1D* in patients with ASD [23]. The role of other VGCC-encoding genes, including *CACNA1I* and *CACNA1S*, in SCZ/ASD remains unexplored, in spite of genetic evidence supporting the contribution of these loci to SCZ/ASD in WESs, GWASs, and genetic association studies [5, 27–29]. Furthermore, how these genetic variations associated with SCZ/ASD directly alter biophysical channel properties and causally affect SCZ/ASD pathophysiology remains unknown.

To address these issues, we report here a genetic and biological study that sought to discover possible disease-associated, rare, single-nucleotide variants (SNVs) with potentially damaging effects, in four VGCC-encoding genes, including *CACNA1C, CACNA1D, CACNA1I*, and *CACNA1S*. We first sequenced the exonic regions of *CANCA1C, CANCA1D, CANCA1I*, and *CACNA1S* in Japanese patients with SCZ/ASD, and then performed association studies for prioritized variants to detect rare, potentially disruptive SNVs. Our analysis identified a new *de novo* SNV, p.A36V *CANCA1C*, in a SCZ patient. We investigated the functional consequence of this mutation on the voltage-gated calcium channel, via both *in-silico* three-dimensional (3D) structure prediction modeling and electrophysiological patch-clamp recording. The neuronal splicing isoform of Ca_v_1.2 with the p.A36V mutation showed reduced Ca^2+^-dependent inhibition, presumably resulting in excessive Ca^2+^ entry through the mutant channel. Thus, our analysis successfully identified an ultra-rare and functionally significant variant in VGCC genes, supporting the hypothesized role of these loci in the risk of SCZ/ASD.

## Methods

### Subjects

Three independent sample sets were used in this study. The first set, comprising samples from 370 patients with SCZ (mean (±SD) age = 49.73 ± 14.75 years; males = 52.97%) and 192 patients with ASD (mean age = 16.34 ± 8.36 years; males = 77.60%), was sequenced for rare point mutations. The second, larger set, comprising samples from 1871 patients with SCZ (mean age = 48.59 ± 14.12 years; males = 51.84%), 380 patients with ASD (mean age = 18.97 ± 9.89 years; males = 78.48%), and 1916 controls (mean age = 44.95 ± 15.16 years; males = 52.07%), was used for association analysis of selected variants detected in the first set. For further analysis of variants detected in the first and second set, we included an independent sample set composed of 2398 patients with SCZ (mean age = 52.06 ± 13.63 years; males = 58.55%) and 3679 controls (mean age = 40.60 ± 13.42 years; males = 39.14%).

All participants in this study were recruited in the Nagoya University Hospital and its associated Institutes. Patients were included in the study if they met DSM-5 criteria for SCZ or ASD and were physically healthy. Healthy controls were selected from the general population and had no personal or family history of psychiatric disorders (first-degree relatives only, based on an interview with the subject). Selection was based on the following: questionnaire responses from the subjects themselves during the sample inclusion step, or an unstructured diagnostic interview conducted by an experienced psychiatrist during the blood collection step. All subjects were unrelated, lived in the central area of the Honshu island of Japan, and self-identified as members of the Japanese population. The Ethics Committees of the Nagoya University Graduate School of Medicine and Research Institute of Environmental Medicine approved this study. All experiments were performed in accordance with the Committee’s guidelines and regulations. Written informed consent was obtained from all participants. In addition, each patient’s capacity to provide consent was confirmed by a family member when needed. Individuals with a legal measure of reduced capacity were excluded.

### Resequencing and data analysis

Genomic DNA was extracted from whole blood or saliva using the QIAGEN QIAamp DNA blood kit or tissue kit (QIAGEN, Hilden, Germany). Custom amplification primers were designed, using Ion AmpliSeq Designer (Thermo Fisher Scientific, Waltham, MA, USA), to cover coding exons and flanking intron regions of the selected genes. Sample amplification and equalization were achieved using Ion AmpliSeq Library Kits 2.0 and the Ion Library Equalizer Kit, respectively (Thermo Fisher Scientific). Amplified sequences were ligated with Ion Xpress Barcode Adapters (Thermo Fisher Scientific). Emulsion PCR and subsequent enrichment were performed using the Ion OneTouch Template Kit v2.0 on Ion OneTouch 2 and Ion OneTouch ES, respectively (Thermo Fisher Scientific). The final product then was sequenced on the Ion PGM sequencing platform (Thermo Fisher Scientific). Raw data output from the sequencer was deposited in the DNA Data Bank of Japan (DDBJ) (http://www.ddbj.nig.ac.jp) under Accession Number DRA004490, and uploaded to the Torrent Server (Thermo Fisher Scientific) for variant calling, with NCBI GRCh37 as a reference. The resulting VCF (variant call format) files were analyzed by Ingenuity Variant Analysis (QIAGEN) for annotation and visualization. Combined Annotation Dependent Depletion (http://cadd.gs.washington.edu/) was applied for annotation of genetic variants.

### Prioritization and association analysis

Missense mutations, small insertions/deletions, and splicing site variations with a minor allele frequency <1% were selected from the annotated data. The mutation calls then were validated for confidence by Sanger sequencing using the BigDye Terminator v3.1 Cycle Sequencing Kit (Thermo Fisher Scientific). Genotyping prioritization was based on whether the mutation was 1) located in a functional domain or motif of the protein, according to the Human Protein Reference Database (http://www.hprd.org), Pfam (http://pfam.xfam.org/), and existing literature, or functionally important, such as causing a frame shift, stop gain, or cysteine gain/loss; 2) a rare variant with a minor allele frequency <1%, or novel or registered in the NCBI dbSNP database (Build 137) (http://www.ncbi.nlm.nih.gov/SNP/), the 1000 Genomes Project (http://www.1000genomes.org/), the Exome Variant Server of NHLBI GO Exome Sequencing Project (ESP6500SI-V2) (http://evs.gs.washington.edu/EVS/), the Human Genetic Variation Database of Japanese genetic variation consortium (http://www.genome.med.kyoto-u.ac.jp/SnpDB), or the Integrative Japanese Genome Variation database (https://ijgvd.megabank.tohoku.ac.jp/); and 3) predicted to represent a nonsynonymous deleterious mutation by at least one of five *in silico* algorithms (PolyPhen2 HumDiv and HumVar [30], LRT [31], MutationTaster [32], and SIFT [33]). Additional *in silico* analysis, including conservation status, was performed for the prioritized SNVs using HomoloGene (http://www.ncbi.nlm.nih.gov/homologene).

Custom TaqMan SNP (single nucleotide polymorphism) genotyping assays were designed and ordered from Applied Biosystems. Allelic discrimination analysis was performed on an ABI PRISM 7900HT Sequence Detection System (Thermo Fisher Scientific). Allele and genotype frequencies of the mutations were compared between patients with SCZ and controls, or between patients with ASD and controls, using Fisher’s exact test (two-tailed), with a threshold of significance set at p < 0.05.

### Identification of de novo SNVs

We examined the inheritance pattern of the prioritized SNVs if parental samples were available. For cases with *de novo* SNVs, we performed paternity testing using an Identifiler plus kit for 15 short tandem repeats (AmpFlSTR Identifiler plus kit, Thermo Fisher Scientific), and each parental genotype was consistent with each of their child’s genotypes. The amplicons were loaded using an ABI 310 Genetic Analyzer (Thermo Fisher Scientific) and genotyped automatically using GeneMapper ID v3.2 software (Thermo Fisher Scientific). The probabilities for calculated paternity supported the conclusion that parental samples were collected from the proband’s biological parents. Among parents of cases with *de novo* SNVs, all were psychiatrically healthy based on self-reporting or questionnaire responses.

### Plasmid construction and mutagenesis

Human brain and human cerebral cortex total RNA (Takara Bio, Kusatsu, Japan) or human heart total RNA (Takara Bio) was reverse-transcribed using SuperScript IV Reverse Transcriptase (Thermo Fisher Scientific). Human *CACNA1C* isoforms were amplified by PCR using Platinum SuperFi DNA polymerase (Thermo Fisher Scientific) and the following primer pairs: the short and neuronal *CACNA1C* isoform was amplified using forward, CTCGAGCTCAAGCTTGCCACCATGCTTCGAGCCTTTGTTCAG, and reverse, CGGGCCGGTACCCTACAGGCTGCTGACGTAGA; the long and cardiac *CACNA1C* isoform was amplified using forward, CTCGAGCTCAAGCTTGCCACCATGCTTCGAGCCTTTGTTCAG, and reverse, CGGGCCGGTACCCTACAGGCTGCTGACGTAGACCCT. The resulting PCR fragment was inserted into a pCAG vector [34]. The clones prepared from human brain and cerebral cortex total RNA were identified as *CACNA1C* isoform 14 in the RefSeq database (NM_001129840.1) and used as neuronal (exon 1-containing short) isoform in this study. The clones from human heart total RNA were identified as *CACNA1C* isoform X30 (XM_006719017.2) and used as cardiac (exon 1a-containing long) isoform in this study. We introduced the point mutation corresponding to p.A36V into the wild-type (WT) *CACNA1C* by the megaprimer method [35]. The first PCR was performed with the aforementioned forward primer used for *CACNA1C* cloning, and a reverse primer (GGGCCAGCCCCACTGCCGCATTG) containing the mutation corresponding to p.A36V. The second PCR was performed with the first PCR product (megaprimer) and the reverse primer used for *CACNA1C* cloning, and the resulting PCR product was inserted into the pCAG vector. Following construction, the identities of plasmid inserts were confirmed by sequencing.

### Immunoblotting

For immunoblotting experiments, HEK293T cells were maintained in Dulbecco’s Modified Eagle’s Medium (DMEM; Nacalai Tesque, Kyoto, Japan) supplemented with 10% (vol/vol) heat-inactivated fetal bovine serum (FBS; Merck, Darmstadt, Germany) and Penicillin-Streptomycin Mixed Solution (Nacalai Tesque) under 5% CO_2_ in air at 37 °C. Cells were transfected in 35-mm tissue culture dishes (IWAKI, Kawajiri, Japan) with 1 µg of the pCAG-*CACNA1C*-WT-IRES-EGFP (WT-Ca_v_1.2) or pCAG-*CACNA1C*-A36V-IRES-EGFP (A36V-Ca_v_1.2) plasmids using X-tremeGENE 9 DNA Transfection Reagent (Roche, Basel, Germany) according to the manufacturer’s instructions. Immunoblotting experiments were performed at 28 hours after transfection.

Transfected HEK293T cells were collected with ice-cold phosphate-buffered saline (PBS) and sonicated in RIPA buffer (50 mM Tris-HCl, pH 7.5, 150 mM NaCl, 0.5% sodium deoxycholate, 0.1% sodium dodecyl sulfate (SDS), 1% NP-40) supplemented with protease inhibitor cocktail (Nacalai Tesque). After centrifugation for 5 min at 15,000 × *g*, the supernatant was collected and combined with 6× Sample buffer (Nacalai Tesque); the mixture then was denatured at 90 °C (for immunoblotting of β-actin) or at room temperature (for immunoblotting of Ca_v_1.2). The extracted proteins were separated on 12% (β-actin) or 6% (Ca_v_1.2) SDS-polyacrylamide gels and transferred onto polyvinylidene fluoride membranes (Merck). The membranes were blocked with 5% skim milk (Nacalai Tesque) in Tris-buffered saline containing 0.05% Tween-20 (TBS-T), incubated with primary antibodies diluted in 2.5% skim milk in TBS-T overnight, and washed with TBS-T. The primary antibodies were detected with horseradish peroxidase (HRP) -conjugated secondary antibodies and ECL Prime Western Blotting Detection Reagent (GE Healthcare, Piscataway, NJ, US). Images were captured using a ChemiDoc Touch Imaging System (Bio-Rad, Hercules, CA, USA). The antibodies used for immunoblotting were purchased commercially as follows: a rabbit polyclonal antibody against Ca_v_1.2 (ACC-003, Alomone Labs, Jerusalem, Israel), a mouse monoclonal antibody against β-actin (A5441, Merck), and secondary antibodies conjugated to HRP (ab97051 and ab97023, abcam, Cambridge, UK).

### Immunocytochemistry

For immunocytochemistry, Baby Hamster Kidney (BHK) cells were cultured in 24-well plastic tissue culture plates (IWAKI) containing 12 mm circular cover glasses (Glaswarenfabrik Karl Hecht, Sondheim vor der Rhön, Germany) coated with poly-L-lysine (Merck); cells were cultured in DMEM (Nacalai Tesque) supplemented with 10% (vol/vol) heat-inactivated FBS and Penicillin-Streptomycin Mixed Solution under 5% CO_2_ in air at 37 °C. Cells were transfected in 24-well plastic tissue culture plates (IWAKI) with 0.2 µg pCAG-α_2_δ-P2A-β_3_-T2A-mCherry-KRasCT [34] and 0.05 µg pCAG-*CACNA1C*-WT or pCAG-*CACNA1C*-A36V plasmids using X-tremeGENE 9 DNA Transfection Reagent. After 24 hours, transfected cells were fixed for 15 min with PBS containing 4% paraformaldehyde, permeabilized for 10 min with PBS containing 0.2% Triton X-100, and incubated for 60 min with 5% normal goat serum and 1% bovine serum albumin in PBS to block nonspecific antibody binding. After pretreatment, cells were incubated overnight with rabbit polyclonal anti-Ca_v_1.2 antibody (1:1000, Alomone Labs) in blocking solution, and then stained for 1 hour with an AlexaFluor 488-conjugated anti-rabbit IgG goat antibody (1:1000, Thermo Fisher Scientific) and Hoechst 33342 (Thermo Fisher Scientific) in PBS. All images were captured with a LSM 710 confocal microscope (Cael Zeiss Microscopy, Jena, Germany) equipped with a 20×/0.8 numerical aperture (NA) objective. To evaluate Ca_v_1.2 trafficking to the plasma membrane, we defined the regions of interest (ROIs) of the plasma membrane based on membrane-tethering red fluorescent protein (RFP-KRasCT) images using an intensity threshold. Cytoplasmic ROIs were defined as inside regions of the plasma membrane ROIs excluding nucleus regions. After background subtraction, mean Ca_v_1.2 intensity from each ROI was used to calculate Ca_v_1.2 intensity ratio (membrane/cytoplasm). Statistical comparison between WT and A36V mutant channels was performed by two-tailed Welch’s t test.

### Modeling of the 3D structure of the Ca_v_1.2 calcium channel and its N-terminus

We performed homology modeling of Ca_v_1.2 using three-dimensional (3D) structures homologous to Ca_v_1.2, as obtained from the Protein Data Bank (PDB; version 2021/11/17) using PSI-BLAST [36] with the help of the HOMCOS server [37]. We focused here on three structures: the rabbit Ca_v_1.1 channel (PDB ID:5gjw; 78% protein sequence identity to Ca_v_1.2) [38], the cockroach Na_v_ channel (PDB ID:6a95; 32% protein sequence identity to Ca_v_1.2) [39], and a complex of CaM and the N-terminal spatial Ca^2+^-transforming element (NSCaTE)region of Ca_v_1.2 (PDB ID: 2lqc; 100% protein sequence identity to Ca_v_1.2) [40]. Individual homologous template 3D structures are shown in Fig. S3, and the overall aligned regions are shown graphically in Fig. S1. Because the most similar structure (5gjw) does not cover the mutated residue 36, we employed a chimeric template. We observed that the conformations of these structures were inconsistent in the N-terminal region; notably, the conformation of the NSCaTE region is α-helical in 2lqc, but assumes a β-sheet-like structure in 6a95 (see Figs. S2 and S3). Given that the N-terminal region (amino acids 1-89) was predicted to form an intrinsically disordered region using DISOPRED 3.16 [41], we hypothesized that the N-terminal region might be able to assume several different conformations, and therefore decided to build models of two different structures: a folded N-terminal structure and a CaM-binding structure. The template of the folded N-terminal structure was generated by combining the structure 5gjw and the N-terminal structure of 6a95 (Figs. S1, S3b). The template of the CaM-binding structure also was generated by combining the structure 5gjw and the N-terminal structure of 6a95, but in this case the NSCaTE region (47-68) was taken from 2lqc chain B (Figs. S1, S3c). The combination of several structures was performed using MATRAS [42] and an in-house Python script. Using the two hybrid template structures, we successfully built two distinct models of Ca_v_1.2 using MODELLER 10.2 [43]. To refine the 3D structure of the model, we repeated short molecular dynamics simulations of N-terminal domain (31-111 residues) of the 3D model using AMBER 20 [44] to generate 10 final structures, and chose one representative from these ten structures (Fig. S4). Details of the modeling are described in Supplementary information. The model of the folded N-terminal structure is shown in Fig. 2a, and that of the CaM-binding structure is shown in Fig. 2b.

### Electrophysiology

For electrophysiology, BHK cells stably expressing α_2_δ and β_2_ subunits (BHK-α_2_δ + β_2_), described previously [45], were used. BHK-α_2_δ + β_2_ cells were cultured in DMEM (Merck) supplemented with 10% (vol/vol) heat-inactivated FBS (Thermo Fisher Scientific), 30 unit/mL penicillin (Meiji Seika Pharma, Tokyo, Japan), and 30 μg/mL streptomycin (Meiji Seika Pharma) under 5% CO_2_ in air at 37 °C. BHK-α_2_δ + β_2_ cells were seeded into Primaria™ 60-mm cell culture dishes (Corning, Glendale, AZ, USA) and grown to 80–90% confluence. The BHK-α_2_δ + β_2_ cells were used between passages 2 and 10. DNA electroporation was performed with a MicroPorator MP-100 (NanoEnTek, Seoul, South Korea) with 10-μL tips according to the manufacturer’s guidelines. For each electroporation, 1 × 10^5^ BHK-α_2_δ + β_2_ cells were suspended in Buffer R and mixed with 0.5 μg WT- or A36V-*CACNA1C* plasmid and 0.08 μg EGFP-N1 (Takara Bio) vector as the marker for the transfected cells. The electroporation settings were 1,150 V, 20 ms, and 2 pulses. Following electroporation, the cells were transferred into Primaria™ 35-mm cell culture dishes (Corning). Electrophysiological experiments were performed at 3–5 days after electroporation.

Currents were recorded using whole-cell patch-clamp techniques [46] with an EPC-10 patch-clamp amplifier (HEKA Elektronik, Reutlingen, Germany) at room temperature. Patch pipettes were made from borosilicate glass capillaries (outer diameter, 1.5 mm; Hilgenberg, Malsfeld, Germany) using a Model P-87 Flaming-Brown micropipette puller (Sutter Instrument, Novato, CA, USA). Pipette resistance ranged from 2 to 3.5 Mohm when filled with the pipette solution described below. The series resistance was electronically compensated to 60% and both the leakage and the remaining capacitance were subtracted by -P/5 method. Currents were sampled at 20 kHz after low-pass filtering at 2.9 kHz. The pipette solution contained the following components (in mM concentrations): Cs-aspartate 95, CsCl 40, MgCl_2_ 4, ATP-Na_2_ 2, phosphocreatine-Na_2_ 10, EGTA 5, and HEPES 5, and was adjusted to pH 7.2 with CsOH. The pipette solution for Ca^2+^-dependent inactivation (CDI) recording contained (in mM concentrations): Cs-methanesulfonate 130, CsCl 5, MgCl_2_ 5, ATP-Na_2_ 2, phosphocreatine-Na_2_ 5, EGTA 0.5, and HEPES 10, and was adjusted to pH 7.2 with CsOH. The ‘10 mM Ba^2+^’ external solution contained the following components (in mM concentrations): tetraethylammonium (TEA) -Cl 140, BaCl_2_ 10, glucose 10, and HEPES 10, and was adjusted to pH 7.4 with TEA-OH. The ‘10 mM Ca^2+^’ external solution contained the following components (in mM concentrations): TEA-Cl 140, CaCl_2_ 10, glucose 10, and HEPES 10, and was adjusted to pH 7.4 with TEA-OH. Osmolarity of all solutions was approximately 300 mOsm. Rapid exchange of the external solutions surrounding the cell was provided within 1 sec by a modified ‘Y-tube’ method [47].

The current–voltage (*I*–*V*) relationship was fitted with the following equation: *I* (*V*_m_) = G * (E_rev_ − *V*_m_)/(1 + exp((V_0.5_ − V_m_)/k), where *I* (*V*_m_) is the peak Ba^2+^ current at the membrane potential of V_m_, G is the maximum conductance, E_rev_ is the apparent zero current potential in the *I*–*V* relationship, V_0.5_ is the potential to give a half-value of conductance, and k is the slope factor that determines the steepness of the curve. To examine the voltage-dependency of inactivation, currents were evoked by 20-ms test pulses to 20 mV after 10-ms repolarization to −80 mV following 2-s conditioning membrane potential displacement from −80 to 10 mV with 10-mV increments. Currents were recorded every 30 s. The curves were fitted using the Boltzmann’s equation, 1/(1 + exp((V_m_ − V_0.5_)/k), where V_m_ is the conditioning membrane potential, V_0.5_ is the potential to give a half-inactivation of conductance, and k is the slope factor that determines the steepness of the curve. Statistical comparison between WT and A36V mutant channels was performed by a two-tailed non-paired Student’s t test.

## Results

### Sequencing and association study

After sequencing of the *CACNA1C, CANCA1D, CANCA1I*, and *CACNA1S* coding regions and the prioritization of sequencing data, we identified several potentially interesting variants, including 1 rare nonsense SNV (p.C1451* in *CACNA1D*), 1 rare short deletion (p.E1675del in *CACNA1D*), and 14 rare NSstrict-damaging SNVs (Table 1). All mutations were confirmed using Sanger sequencing, and all of these alleles were heterozygous. Our sequence data are available in the DDBJ (http://www.ddbj.nig.ac.jp) as Accession Number DRA004490DNA. Among the identified mutations, potentially highly disruptive variants (including p.A36V in *CACNA1C*, p.C1451* in *CACNA1D*, p.E1675del in *CACNA1D*, and NSstrict SNV p.G330R in *CACNA1D*) were genotyped in an independent sample set comprising 1871 SCZ cases, 380 ASD cases, and 1916 controls (Table 2). Neither p.A36V in *CACNA1C* nor p.C1451* in *CACNA1D* were found in SCZ/ASD cases or healthy controls in the independent sample set, suggesting that these SNVs might be ultra-rare SNVs in the Japanese population. p.E1675del *CACNA1D* was found in both SCZ/ASD cases and healthy controls, but frequencies in psychiatric patients and healthy controls did not exhibit a statistically significant difference. p.G330R *CACNA1D* was found in SCZ/ASD cases, but not in healthy controls, suggesting that this mutation might constitute a significant SNV that is enriched exclusively in patients with psychiatric disorders. Therefore, an additional association study was performed in a much larger independent sample set comprising 4255 SCZ cases and 5565 controls. This analysis revealed that p.G330R *CACNA1D* was found in both SCZ cases and healthy controls, but frequencies in psychiatric patients and healthy controls did not exhibit a statistically significant difference (Table 3).

**Table 1:** Details of potentially functional damaging variants after the prioritization of resequencing data.

									*in-silico* analyses of protein function prediction					Evolutionary conservation score
Type	Chromosome ^b^	Position ^b^	Reference allele	Sample Allele	Gene	Protein Variant	dbSNP^c^	Phenotype^d^	SIFT	PolyPhen-2 HumanVar	PolyPhen-2 HumanDiv	Mutation Taster	LRT	Primate PhastCons^e^
Rare disruptive	3	53810063	T	A	CACNA1D	p.C1451*		ASD						0.994
Rare indel	3	53834368	AGA		CACNA1D	p.E1675del	rs778776240	SCZ/ASD						0.976
Rare NSstrict^a^	1	201028331	C	T	CACNA1S	p.A1171T		SCZ	Damaging	Probably Damaging	Probably Damaging	disease causing	damaging	0.997
	1	201031171	A	G	CACNA1S	p.L985P		SCZ/ASD	Damaging	Probably Damaging	Probably Damaging	disease causing	damaging	0.034
	1	201034980	C	T	CACNA1S	p.V947I	rs76460090	SCZ/ASD	Damaging	Probably Damaging	Probably Damaging	disease causing	damaging	0.923
	1	201036037	A	G	CACNA1S	p.S879P	rs573597311	SCZ/ASD	Damaging	Probably Damaging	Probably Damaging	disease causing	damaging	0.997
	1	201036040	C	T	CACNA1S	p.V878M	rs202131129	SCZ	Damaging	Probably Damaging	Probably Damaging	disease causing	damaging	0.998
	1	201043740	G	T	CACNA1S	p.L653I		SCZ	Damaging	Probably Damaging	Probably Damaging	disease causing	damaging	0.957
	1	201046194	A	G	CACNA1S	p.S561P		SCZ	Damaging	Probably Damaging	Probably Damaging	disease causing	damaging	0.998
	1	201047034	C	T	CACNA1S	p.R531H	rs748711395	SCZ	Damaging	Probably Damaging	Probably Damaging	disease causing	damaging	0.998
	1	201058429	T	C	CACNA1S	p.Y286C		SCZ	Damaging	Probably Damaging	Probably Damaging	disease causing	damaging	0.998
	3	53700434	G	C	CACNA1D	p.G330R		SCZ	Damaging	Possibly Damaging	Possibly Damaging	disease causing	damaging	0.926
	3	53760951	A	G	CACNA1D	p.M736V	rs775056182	SCZ	Damaging	Possibly Damaging	Probably Damaging	disease causing	damaging	0.993
	3	53760987	G	A	CACNA1D	p.V748I	rs184573217	SCZ/ASD	Damaging	Possibly Damaging	Probably Damaging	disease causing	damaging	0.989
	12	2775874	G	T	CACNA1C	p.V1545L		SCZ	Damaging	Probably Damaging	Probably Damaging	disease causing	damaging	0.981
	22	40066215	G	T	CACNA1I	p.R1456L	rs766713729	SCZ	Damaging	Probably Damaging	Probably Damaging	disease causing	damaging	0.998
De novo	12	2224447	C	T	CACNA1C	p.A36V	rs755028000	SCZ	Tolerated	Benign	Possibly Damaging	disease causing	Neutral	0.06

^a^Rare Nsstrict: Rare nonsynoumous strict-damaging SNVs

^b^Genomic position is based on GRCh37/hg19

^c^dbSNP: dbSNP build 153 (https://www.ncbi.nlm.nih.gov/snp/)

^d^ASD = discovered in autism patients; SCZ = discovered in schizophrenia patients; SCZ/ASD = discovered in schizophrenia and autism patients

^e^Primate PhastCons: Primate PhastCons conservation score (http://compgen.cshl.edu/phast/phastCons-HOWTO.html)

**Table 2:** Association study of selected variants.

Type	Chromosome^b^	Position^b^	Gene	Protein Variant	Schizophrenia			Autism spectrum disorder			Healthy control
					Genotype Count^c^	*P* value	Odds ratio	Genotype count^c^	*P* value	Odds ratio	Genotype count^c^
Rare disruptive	3	53810063	CACNA1D	p.C1451	0/0/1871			0/0/380			0/0/1916
De novo	12	2224447	CACNA1C	p.A36V	0/0/1871			0/0/380			0/0/1916
Rare indel	3	53834368	CACNA1D	p.E1675del	0/3/1868	0.6452	1.024	0/3/377	0.06093	5.069	0/3/1913
Rare NSstrict^a^	3	53700434	CACNA1D	p.G330R	0/2/1869	0.244	inf	0/1/379	0.1655	inf	0/0/1916

^a^Rare Nsstrict: Rare nonsynoumous strict-damaging SNVs

^b^Genomic position is based on GRCh37/hg19

^c^Genotype count: homozygote of minor allele/heterozygote/homozygote of major allele

**Table 3:** Association study of selected variant in the third and largest independent sample set.

Type	Chromosome^a^	Position^a^	Gene	Schizophrenia				Autism spectrum disorder			Healthy control
				Protein Variant	Genotype Count^b^	*P* value^c^	Odds ratio^c^	Genotype count^b^	*P* value^c^	Odds ratio^c^	Genotype count^b^
Rare NSstrict	3	53700434	CACNA1D	p.G330R	0/3/4252	0.2196	0.2547	0/1/379	0.1238	0.0681	0/1/5564

^a^Genomic position is based on GRCh37/hg19

^b^Genotype count: homozygote of minor allele/heterozygote/homozygote of major allele

^c^*P* value and Odds Ratio was calculated by Fisher exact test (one-tail)

### Identification of a de novo SNV

For the rare and potentially function-damaging variants discovered in our sequencing study, we attempted a check of their inheritance pattern if parent or sibling samples were available. We successfully discovered one *de novo* SNV (p.A36V in *CACNA1C*) that was present only in the proband (i.e., parents or siblings did not carry the mutation). The patient with the *de novo* SNV (p.A36V in *CACNA1C*) was diagnosed with SCZ at the age of 23. In the outpatient clinic, the patient showed additional symptoms of SCZ, including irritability and soliloquy. The symptoms were controlled well by risperidone (4 mg per day). The patient was not diagnosed with any diseases other than SCZ. This individual’s electrocardiogram test was normal. The patient had a sibling who also was diagnosed with SCZ, although we lack detailed clinical information for this individual; neither parent exhibited any psychiatric disorder.

### The p.A36V mutation does not show apparent deficits in membrane trafficking by Ca_v_1.2 channels

**Fig. 1:**
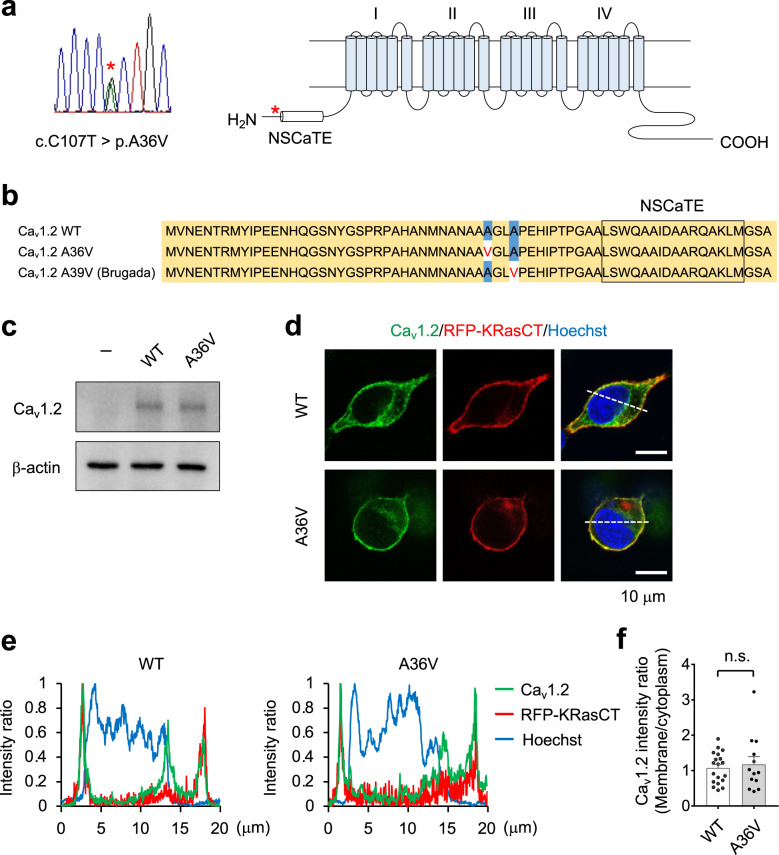
The A36V mutation does not affect membrane localization of Ca_v_1.2 channels. **a** Sanger sequencing results for the *de novo* variant p.A36V (left) and schematic illustration of the primary structure of the Ca_v_1.2 channel (right). The red asterisk indicates the A36V mutation near the N-terminal spatial Ca^2+^-transforming element (NSCaTE). **b** The N-terminal amino acid sequences for Ca_v_1.2 channels (short isoforms). The A36V mutation and the A39V Brugada mutation are indicated in red letters. **c** Expression of wild-type (WT) and A36V Ca_v_1.2 channels in HEK293T cells as detected by anti-Ca_v_1.2 antibody. **d** Membrane localization of WT and A36V Ca_v_1.2 channels overexpressed in BHK cells. The plasma membrane was visualized by membrane-tethering red fluorescent protein (RFP-KRasCT). **e** The fluorescence intensity profiles of the line shown in Fig. 1d. **f** Plasma membrane to cytoplasm intensity ratio of Ca_v_1.2. Statistical comparison was performed by two-tailed Welch’s *t* test (n.s., not significant). Data are presented as mean ± s.e.m.

To assess the biological significance of the A36V mutation in Ca_v_1.2 channels expressed in the brain, we constructed an A36V Ca_v_1.2-encoding plasmid vector. The *CACNA1C* gene includes alternative exons designated exon 1 (short and neuronal) and exon 1a (long and cardiac); we cloned a ‘short’ isoform cDNA containing the alternative exon 1 from adult human brain total RNA. Six clones were obtained in total, and all clones were identified as transcription variant 14 in the RefSeq database (NM_001129840.1). We introduced the mutation encoding the A36V substitution into the plasmid encoding this neuronal isoform, which typically would be expressed predominantly in adult human brain (Fig. 1c). An A39V mutation, located in the vicinity of A36V (Fig. 1a, b) in Ca_v_1.2 channels, previously was linked to Brugada syndrome, which presents with atrial fibrillation and abbreviated QT interval. A cardiac ‘long’ isoform of Ca_v_1.2 containing both the alternative exon 1a and the A39V mutation has been shown to induce a membrane trafficking defect and to exhibit drastically decreased Ca^2+^ currents [48]. Therefore, we examined whether membrane trafficking of neuronal Ca_v_1.2 channels harboring the A36V mutation is defective. When WT and A36V Ca_v_1.2 neuronal channels were expressed with α_2_δ and β_3_ auxiliary subunits in BHK cells, we found that the two forms accumulated to similar levels at the plasma membrane regions (Fig. 1d–f). This result is consistent with a prior observation that A39V Brugada mutation in the neuronal Ca_v_1.2 isoform did not exhibit apparent membrane trafficking deficits [49]. These data suggested that a general loss of membrane expression is unlikely to account for the neuronal phenotype of the *de novo* A36V mutation in Ca_v_1.2.

### The A36V mutation may alter conformational equilibrium between folded and CaM-binding structures at the Ca_v_1.2 N-terminus, thereby modulating CDI

**Fig. 2:**
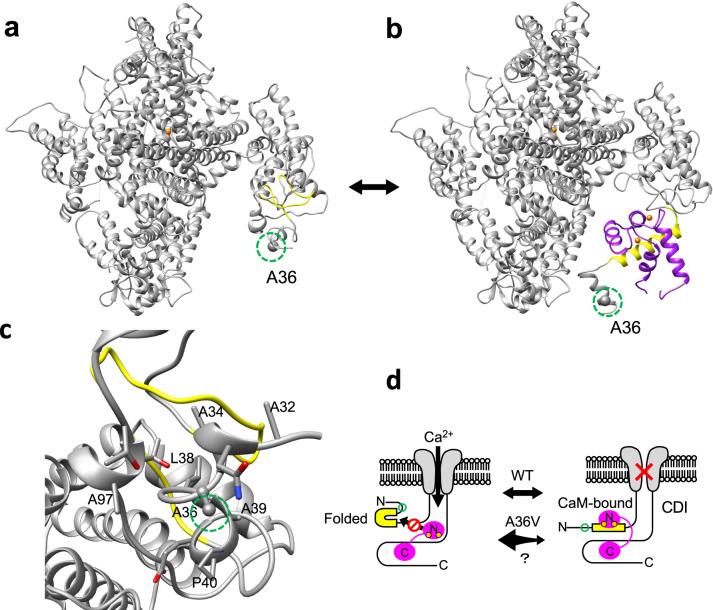
Conformational equilibrium between the folded N-terminal structure and calmodulin (CaM) -binding structure of Ca_v_1.2. **a** Three-dimensional (3D) model structure of the Ca_v_1.2 channel with the folded N-terminal structure. **b** 3D model structure of the Ca_v_1.2 channel with the CaM-binding structure, shown in complex with the N-lobe of CaM. **c** Enlarged view around the alanine 36 (A36) residue of the folded N-terminal structure. The A36 site is highlighted by green dotted circles. The N-terminal spatial Ca^2+^-transforming element (NSCaTE) region (47–68), Ca^2+^, and CaM are indicated in yellow, orange, and magenta, respectively. Molecular graphics were created using UCSF Chimera [74]. **d** A schematic illustration of the hypothesis that the A36V mutation attenuates Ca^2+^-dependent inactivation (CDI) by conformational equilibrium shift favoring the folded structure.

We next tested whether the A36V mutation modulated the biophysical properties of the Ca_v_1.2 protein via alteration of structural dynamics of the alpha1 subunit conformations. Ca_v_1.2 has multiple CaM-binding sites in its N-terminus and C-terminus. Ca^2+^ ions entering through Ca_v_1.2 pore bind to CaM, and consequently cause CDI [50–53]. We noticed that the A36V mutation is located close to the NSCaTE region, a CaM interaction site that has been shown to be necessary for N-lobe dependent CDI [50, 53]. Using homologous 3D-structures, we modeled two different structures of Ca_v_1.2 (a folded N-terminal structure and an open CaM-binding N-terminal structure), and assumed the conformational ensemble of Ca_v_1.2 is in an equilibrium among various structures including these two structures (Fig. 2). The 36^th^ residue of Ca_v_1.2 (alanine in the WT) have hydrophobic contacts with other nonpolar residues (A39 and A97) in the folded N-terminal structure (Fig. 2a, c), whereas this residue is more exposed to solvent in the CaM-binding structure (Fig. 2b). Because valine is more hydrophobic than alanine, the models suggested that the A36V substitution may shift the conformational equilibrium toward the folded N-terminal structure, thereby decreasing the frequency of formation of the CaM-binding structure at the NSCaTE region of Ca_v_1.2 (Fig. 2d).

**Fig. 3:**
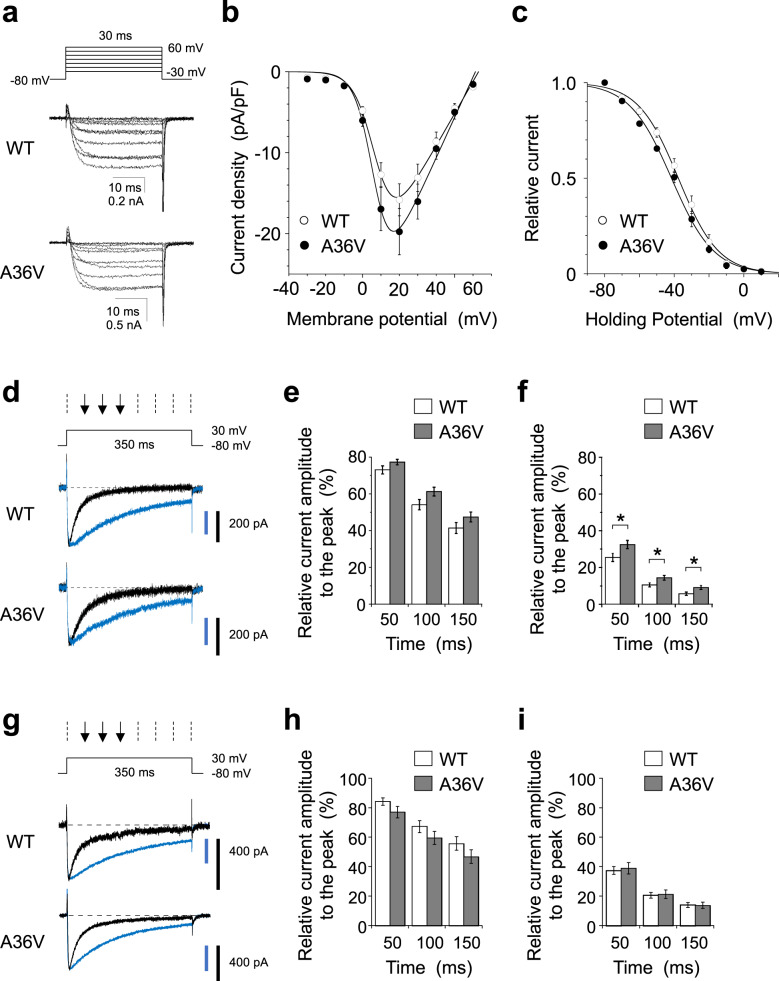
Electrophysiological properties of p.A36V Ca_v_1.2 channels. **a** Families of Ba^2+^ currents evoked by 30-ms depolarizing pulses from −30 to 60 mV with increments of 10 mV for wild-type (WT) and A36V neuronal Ca_v_1.2 channels. **b** Current density–voltage (*I*–*V*) relationships. Data are expressed as mean ± s.e.m., WT: *n* = 18, A36V: *n* = 12. The values of G, Erev, V_0.5_, and k were −0.40, 63.0 mV, 7.6 mV, and 5.6 mV for WT channels, and −0.50, 61.3 mV, 6.7 mV, and 4.9 mV for A36V Ca_v_1.2 channels. **c** Inactivation curves for WT (○, *n* = 9) and A36V (●, *n* = 4) neuronal Ca_v_1.2 channels. Data are expressed as mean ± s.e.m. The values of V_0.5_, and k were (respectively) −37.6 mV and 11.5 mV for WT channels, and −41.6 mV and 12.1 mV for A36V Ca_v_1.2 channels. **d**, **g** Ca^2+^-dependent inactivation (CDI) of neuronal (**d**) and cardiac (**g**) Ca_v_1.2 channels. Ba^2+^ (blue) and Ca^2+^ (black) currents evoked by 350-ms step depolarization to 30 mV were normalized at their peak current amplitudes for WT and A36V Ca_v_1.2 channels. **e**, **f, h, i**, Ratios of current amplitude to the peak amplitude were plotted against depolarizing time in the Ba^2+^ (**e, h**) and the Ca^2+^ (**f, i**) external solutions. The numbers of recorded cells were 10 and 15 for WT and A36V neuronal Ca_v_1.2 channels (**e**, **f**), and 8 and 6 for WT and A36V cardiac Ca_v_1.2 channels (**h**–**i**), respectively. Statistical comparison was performed by two-tailed non-paired Student’s *t* test (*p < 0.05). Data are presented as mean ± s.e.m.

To determine the electrophysiological characteristics of the A36V Ca_v_1.2 channels, we recorded whole-cell Ba^2+^ currents through recombinant Ca_v_1.2 channels transiently expressed in BHK-α_2_δ+β_2_ cells [45]. The current–voltage (I–V) relationship for the neuronal A36V Ca_v_1.2 channel was similar to that for the WT channel. The peak current density (peak current amplitude divided by cell capacitance) for the neuronal A36V Ca_v_1.2 channel (15.8 ± 2.0 pA/pF, *n* = 18) did not differ significantly from that of the neuronal WT Ca_v_1.2 channel (19.8 ± 2.9 pA/pF, *n* = 12) (Fig. 3a, b), consistent with a lack of a mutation effect on channel membrane trafficking (Fig. 1d–f). The steady-state inactivation curves for both channels were very similar (Fig. 3c). These results suggest that the A36V mutation does not change the voltage dependency of activation and inactivation of the neuronal Ca_v_1.2 channel.

Finally, we examined the effect of A36V on global CDI. This parameter was tested with the use of the pipette solution containing 0.5 mM EGTA. We recorded Ba^2+^ and Ca^2+^ currents at 30 mV from the same BHK cell expressing neuronal Ca_v_1.2 (Fig. 3d–f). Ba^2+^ currents decayed slowly, but Ca^2+^ currents decayed completely within 350 msec (Fig. 3d). The ratio of remaining current amplitude to the peak current amplitude was calculated at different time points after depolarization to 30 mV (Fig. 3e, f). Ca^2+^ currents (Fig. 3f), but not Ba^2+^ currents (Fig. 3e) of neuronal A36V Ca_v_1.2, were reduced mildly, but significantly more slowly, than Ca^2+^ currents of the neuronal WT Ca_v_1.2, suggesting that the A36V mutation affects global CDI in neurons. We further examined the impact of A36V in the cardiac isoform that contain the longer N-terminal region encoded by exon 1a (Fig. 3g–i). In contrast, Ca^2+^ currents (Fig. 3i) and Ba^2+^ currents (Fig. 3h) of cardiac A36V Ca_v_1.2 decayed similarly to those of cardiac WT Ca_v_1.2. The reported electrophysiological properties of the A39V Brugada mutation in a neuronal isoform is more complex [54], suggesting co-existing overlapping pathophysiology of the A39V mutation, especially in cardiac mutant channels. Taken together, these data indicated that A36V neuronal Ca_v_1.2 is a mild gain-of-function mutant channel, while the A36V cardiac Ca_v_1.2 behaves similarly to the WT channel, consistent with the relatively late onset of SCZ symptoms and normal cardiac functions.

## Discussion

Recent large-scale genetic studies have reported that ultra-rare disruptive SNVs are highly enriched in patients with SCZ/ASD, especially SNVs in sets of VGCC genes, providing an opportunity to discover the cellular processes that are relevant to the pathogenesis of SCZ/ASD [5, 11, 22, 23, 55]. However, the cellular processes whereby these rare disruptive SNVs give rise to psychiatric disorders remain unknown. To address this question, we performed a comprehensive search, including a genetic and functional analysis of VGCC genes. Our analysis identified 1 nonsense SNV (p.C1451* in *CACNA1D*), 1 *de novo* SNV (p.A36V in *CACNA1C*), 1 rare short deletion (p.E1675del in *CACNA1D*), and 14 NSstrict SNVs (Non-synonymous SNV predicted as damaging by all of five *in silico* analyses). Neither p.A36V *CACNA1C* nor p.C1451* *CACNA1D* were found in 1871 SCZ cases, 380 ASD cases, or 1916 healthy controls in the independent sample set, suggesting that these SNVs might be ultra-rare SNVs in the Japanese population. p.G330R *CACNA1D* was detected in both SCZ and healthy controls, at frequencies that did not show statistically significant differences between the two groups. However, it should be noted that the sample size was too small to completely rule out the possibility of association. The p.G330R mutation is located in the S5-S6 linker of repeat I of the encoded protein; this region previously was shown to be a critical determinant of L-type VGCC conductance [56], suggesting that this mutation may have biological significance. To assess the effect of the A36V mutation on Ca_v_1.2 channel properties, we performed *in silico* 3D structural prediction modeling and electrophysiological studies. The modeling analysis led to a previously unexplored (to our knowledge) hypothesis, that the N-terminal region of Ca_v_1.2 exists in an equilibrium between two alternative conformations, a folded N-terminal structure and an open CaM-binding structure. We postulated that the A36V mutation of neuronal Ca_v_1.2 may shift the conformational equilibrium toward the folded N-terminal structure, thereby modulating the CDI of neuronal Ca_v_1.2 channels. Consistent with this hypothesis, we observed a decrease in the CDI of A36V neuronal Ca_v_1.2 channels. These results support the proposal that the newly identified A36V mutation affects channel properties in a mild gain-of-function fashion, potentially explaining changes in pathophysiology underlying SCZ.

Our analysis identified a new *de novo* SNV, p.A36V *CACNA1C*, in a SCZ patient; we therefore investigated the functional consequence of this mutation on the function of the voltage-gated calcium channel, via both *in silico* 3D structure prediction modeling and electrophysiological patch-clamp recording. Modeling analysis suggested a possible shift in the dynamic equilibrium of A36V Ca_v_1.2’s N-terminus, disfavoring a calmodulin (CaM) -bound structure. Consistent with this modeling, A36V neuronal Ca_v_1.2 showed a significant reduction in CDI compared to WT neuronal Ca_v_1.2, with little change in voltage-dependent activation and inactivation. CDI is a negative feedback mechanism limiting excessive Ca^2+^ entry [51, 57]; therefore, A36V can be regarded as a gain-of-function mutation in neuronal Ca_v_1.2. Thus, our analysis successfully identified an ultra-rare and functionally significant variant in VGCC-encoding genes, supporting the hypothesized role of these loci in the risk of SCZ/ASD, and indicating that a mild gain-of-function in Ca_v_1.2 may play a role in the etiology of SCZ.

A series of GWAS and WES studies have suggested causal links between common and rare variants of *CACNA1C* and psychiatric disorders including SCZ, BD, and ASD [19]. Growing evidence has shown that gain-of-function mutations in Ca_v_1.2 are associated with psychiatric disorders. For instance, a *CACNA1C* lesion causing a G406R substitution in the encoded protein was identified in patients with Timothy syndrome; multiple studies have shown that this gain-of-function mutation is associated with prolonged QT intervals, syndactyly, and ASD [14, 15]. Furthermore, the *CACNA1C* gene polymorphism rs1006737, known to be associated with SCZ and BD, has been shown to augment Ca_v_1.2 channel activity and *CACNA1C* mRNA expression in induced human neurons and in human brain [58, 59]. Intriguingly, the Brugada syndrome A39V Ca_v_1.2 mutation, when encoded in a long splicing isoform (exon 1a-containing cardiac type), was shown to function as a loss-of-function mutation with impaired membrane trafficking [48]; when encoded in a short splicing isoform (exon 1-containing neuronal type), this change possibly functions as a gain-of-function mutation by modulating CDI [54]. These splicing isoform-specific observations suggest that the presence of the same Ca_v_1.2 mutation in separate isoforms leads to distinct outcomes.

The present study showed that the A36V mutation, when carried by the short neuronal isoform, inhibits CDI, suggesting that A36V and A39V, which are located near the NSCaTE domain in Ca_v_1.2, both serve as mild gain-of-function mutations in the neuronal isoform. *In silico* 3D structural modeling indicated that these residues form part of a hydrophobic core in the N-terminal folded structure, but are exposed in the CaM-binding structure (Fig. 2). These substitutions with more hydrophobic residues may stabilize the N-terminal folded structure while destabilizing the CaM-binding structure, which in turn could affect the CDI. Given that the A36V mutation was identified from an individual with SCZ without cardiac symptoms, this mutation may not influence the activity of cardiac channels. Consistent with this notion, it was found that A36V mutation did not affect electrophysiological properties when we tested the mutation in the cardiac isoform. Ca_v_1.2 has multiple isoforms with different functional properties and tissue-selective expression [60, 61]. To understand the complexity of the contribution of A36V and other mutations to psychiatric disorders and cardiac arrhythmia, future studies will need to consider cell type (neuronal or cardiac), Ca_v_1.2 splicing isoform, and co-expressed auxiliary subunits that contribute to the functional diversity of Ca_v_1.2 channels [62].

Trios-based exome sequencing studies have supported the hypothesis that *de novo* SNVs, especially *de novo* SNVs in protein-coding sequences, are related to increased risk for psychiatric disorders such as SCZ/ASD and BD [63–66]. Furthermore, a relationship between advanced paternal age and increased ASD risk has been established in different studies [67], and *de novo* SNVs were indicated as potential risk factors [68]. Notably, a sibling of the A36V carrier identified in the present study also was diagnosed with SCZ; therefore, one or more additional factors (genetic or environmental) may contribute to increased susceptibility to SCZ in this particular family, a conjecture that may be explained, in part, by a two-hit model [69].

Given the potentially important role of *de novo* SNVs in SCZ/ASD susceptibility, we tested for the presence of candidate SNVs in the parents of the carrier patients, if parental DNA samples were available. We discovered one *de novo* missense SNV (p.A36V in *CACNA1C*) in a patient with SCZ, and showed (by electrophysiological recording) that cells expressing the A36V neuronal Ca_v_1.2 channel exhibit slower decreases in Ca^2+^ current than do cells expressing the WT channel. This finding supports the hypothesis that this *de novo* missense SNV is an ultra-rare disruptive SNV affecting CDI, possibly contributing to susceptibility to psychiatric disorders. The Ca^2+^-dependent signaling pathway is a key component in neural circuit formation, gene expression, and neuronal plasticity [70, 71]. In addition to the excessive Ca^2+^ entry per se, disturbance of intracellular Ca^2+^ homeostasis dynamics may lead to neural dysfunction by the dis-regulation of downstream signaling pathways that also are associated with SCZ/ASD [72].

Several limitations should be considered when interpreting the results of our study. First, the lack of statistical significance in our association study may reflect the small sample size; a new study in a larger cohort is needed. Nonetheless, our data revealed that these SNVs are ultra-rare in the Japanese population. These genetic data may be incorporated into future large-scale WESs, potentially supporting the hypothesis that an increased burden of ultra-rare deleterious mutations is observed in patients with SCZ/ASD. Second, due to the lack of enough parental DNA samples, we were able to perform segregation analysis for only a few of the prioritized SNVs. To identify more *de novo* mutations associated with susceptivity to SCZ/ASD, we will need to collect more DNA samples of family members and perform trios-based exome-sequencing studies. Finally, our sequence analysis did not cover the promoters, untranslated regions, or intronic regions of the target genes, which may contain important mutations at regulatory sites.

In conclusion, we identified 1 nonsense SNV (p.C1451* in *CACNA1D*), 1 *de novo* SNV (p.A36V in *CACNA1C*), 1 rare short deletion (p.E1675del in *CACNA1D*), and 14 NSstrict SNVs by genetic analysis of the *CANCA1C, CACNA1D, CACNA1I*, and *CACNA1S* genes in humans. Our genetic and biological data imply that the *de novo* SNV (p.A36V in *CACNA1C*) may increase susceptibility to SCZ pathogenesis by perturbing CDI. Furthermore, our data provide evidence in support of the potential role of VGCC-encoding genes in psychiatric disorders.

## Supplementary information

Supplementary Methods
