# Supplementary material for: Identification of ultra-rare disruptive variants in voltage-gated calcium channel-encoding genes in Japanese samples of schizophrenia and autism spectrum disorder
Source: Transl Psychiatry. 2022 Feb 26;12:84. doi: 10.1038/s41398-022-01851-y (PMC8882172; doi:10.1038/s41398-022-01851-y)
Supplement: Supplementary file 1 — Supplementary Methods [file 41398_2022_1851_MOESM1_ESM.docx]

**Supplementary information**

**Supplementary Methods**

**Modeling of 3D structures of Ca_v_1.2 with and without calmodulin**

The amino acid sequence for the Ca_v_ 1.2 was taken from the RefSeq protein NP_001123312.1, which corresponds to the RNA of the isoform 14 (RefSeq: NM_001129840.1). For homology modeling of Ca_v_1.2, homologous 3D structures for Ca_v_1.2 were searched in Protein Data Bank (version 2021/11/17) using PSI-BLAST [1] with the help of HOMCOS server [2]. A position-specific score matrix of Ca_v_1.2 was generated using vertebrate and invertebrate amino acid sequences of TrEMBL version 2021_04 (25,163,658 sequences) with number of iterations = 5. We focused on three structures; rabbit Ca_v_1.1 channel (PDB ID:5gjw chain A; UniProt ID:CAC1S_RABIT; sequence identity = 78 %) [3], cockroach Na_v_ channel (PDB ID:6a95 chain A; UniProt ID: SCNA1_PARAM; sequence identity = 32 %) [4], and a complex of calmodulin and NSCaTE region of Ca_v_1.2 (PDB ID: 2lqc chain B; UniProt ID: CAC1C_HUMAN; sequence identity = 100 %) [5]. These structures are shown in Fig. S3, and the overall aligned regions are graphically shown in Fig. S1. Because the most similar structure (5gjw) does not cover the mutated 36-th site, we have to employ a chimeric template. However, conformations of these structures are inconsistent in N-terminal region; especially, the conformation of the NSCaTE region is α-helix in the structure 2lqc, whereas β-sheet like structure in the structure 6a95 (see Fig. S2 and S3). Considering N-terminal 1-114 region was predicted as an intrinsically disordered region by DISOPRED 3.16 [6], we assumed that the N-terminal region can have several different conformations and decided to model two different structures: folded N-terminal structure and CaM-binding structure. The template of the folded N-terminal structure was made by combining the structure 5gjw and the N-terminal of 6a95 (Fig. S1 and S3b). The template of the CaM-binding structure was made also by combining the structure 5gjw and the N-terminal of 6a95, but the NSCaTE region (47-68) was taken from 2lqc chain B (Fig. S1 and S3c). Combining several structures was performed by MATRAS [7] and an in-house Python script. Details for preparing templates are described in next paragraphs. Using the two hybrid template structures, we built two models of Ca_v_1.2 by MODELLER 10.3 [8].

The template structure for the folded N-terminal structure was obtained by combining two structures, 5gjw and 6a95. The structure of 6a95 chain A was superimposed on the structure of 5gjw chain A using the protein 3D structure comparison program MATRAS [7]. The superimposed structure of the 93 residues (90-182 of 6a95 chain A, corresponding to 31-123 region of Ca_v_1.1) was extracted and added to the 3D structure of 5gjw chain A to obtain a chimeric 3D template structure (Fig. S1 and S3ab).

The template structure for the CaM-binding structure was generated by three structures: 5gjw, 6a95 and 2lqc. The structure of 6a95 chain A was superimposed on the structure of 5gjw chain A using MATRAS (Fig. S3b). And the superimposed structure of the 33 residues (127-182 of 6a95 chain A, corresponding to 68-123 region of Ca_v_1.1) was extracted and added to the 3D structure of 5gjw chain A. Next, the chain B of 2lqc (corresponding to 45-68 region of Ca_v_1.1) was placed close to the N-terminal of the 6a95 structure by superimposing a pair of three amino acids: “GSA” of 2lqc_B and “AEL” in 6a95_A (see the alignment in Fig. S2). Then 135 degree-rotation was applied to the chain B of 2lqc for avoiding atomic clashes. Calmodulin structure (2lqc chain A) was also transformed together with the chain B of 2lqc. Finally, 90-108 region of 6a95 chain A (corresponds to 31-47 of Ca_v_1.2) was extracted and placed close to the N-terminal of the superimposed 2lqc chain B by superimposing a pair of three amino acids: “HPD” of 6a95_B and “GTG” in 2lqc_B (see the alignment in Fig. S2). Then 225 degree-rotation was applied to the 90-108 region for avoiding atomic clashes. The superimpositions of the three amino acids were performed using an in-house python script. The superimposed structure composed of the four structural fragments with calmodulin was shown in Fig. S3c.

To model the folded N-terminal structure, we used the 90-182 region in 6a95 chain A as the template. But, this template structure is not well-packed (Fig. S3b), and the corresponding N-terminal region of the template-based model is not well-packed either as shown in Fig. S4b. It is because the local resolution of CryoEM 3D map (EMD-6997) for the N-terminal region is not high enough to build a precise atomic structure. To refine the 3D model of the folded N-terminal structure, we performed short molecular dynamics (MD) simulations of 11-101 region of Ca_v_1.1. All simulations used the CPU implementation of sander in AMBER 20 [9] using GBSA implicit solvent model with the combination of GB-Neck2, mbondi3 intrinsic radii and ff14SBonlysc force field. The initial structure was the template-based 3D model of the folded N-terminal structure shown Fig. S4b. After the structure was minimized and heated in 200 psec with positional restraints of main chain atoms, 300 K equilibrating simulations were performed for 2 nsec with positional restraints of the three residues (99-101) of the C-terminal. We repeated the 2 nsec equilibrating simulations ten times using different seed numbers for pseudo-random number generator, by the options ig=1, 2, …, 10. The RMSD values from the initial structures are plotted in Fig. S4a. The final ten structures shown in Fig. S4c-l have various conformations; some conformations such as ig=1,2 are more compact than the initial, but others such as ig=3,4,5 are looser. It consists with the prediction that N-terminal region 11-101 has an intrinsically disorder conformations. Although no single unique conformation was observed, all the NSCaTE regions (47-68) of the ten structures do not have α-helical conformation, it means that calmodulin cannot bind to these ten folded N-terminal structures. In the three structures (ig=2, 8 and 10; Fig. S4djl) of the ten, A36 has hydrophobic contacts with sequentially-separated nonpolar residues, such as A97, L50 and L107. It suggests that the N-terminal region has various non α-helical folded-conformation, and in some of these structures, A36 has hydrophobic interactions, and the mutation A36V can stabilize the folded N-terminal conformation. Among the ten structures, we chose the structure of ig=10 (Fig. S4l) as the representative. Finally, the structure of the 11-101 region generated by MODERLLER was replaced with the representative structure (ig=10; Fig. S4l) generated by the MD simulation, to get the final model of the folded N-terminal structure shown in Fig. 2a.

**References**

1. Altschul SF, Madden TL, Schäffer AA, Zhang J, Zhang Z, Miller W, et al. Gapped BLAST and PSI-BLAST: a new generation of protein database search programs. Nucleic Acids Res. 1997;25:3389–3402.

2. Kawabata T. HOMCOS: an updated server to search and model complex 3D structures. J Struct Funct Genomics. 2016;17:83–99.

3. Wu J, Yan Z, Li Z, Qian X, Lu S, Dong M, et al. Structure of the voltage-gated calcium channel Cav1.1 at 3.6 Å resolution. Nat 2016 5377619. 2016;537:191–196.

4. Shen H, Li Z, Jiang Y, Pan X, Wu J, Cristofori-Armstrong B, et al. Structural basis for the modulation of voltage-gated sodium channels by animal toxins. Science. 2018;362.

5. Liu Z, Vogel HJ. Structural basis for the regulation of L-type voltage-gated calcium channels: interactions between the N-terminal cytoplasmic domain and Ca2+-calmodulin. Front Mol Neurosci. 2012;5:1–46.

6. Jones DT, Cozzetto D. DISOPRED3: precise disordered region predictions with annotated protein-binding activity. Bioinformatics. 2015;31:857–863.

7. Kawabata T, Nishikawa K. Protein Structure Comparison Using the Markov Transition Model of Evolution. Proteins. 2000;41:108–122.

8. Šali A, Blundell TL. Comparative Protein Modelling by Satisfaction of Spatial Restraints. J Mol Biol. 1993;234:779–815.

9. Case DA, Aktulga HM, Belfon K, Ben-Shalom IY, Brozell SR, Cerutti DS, Cheatham III TE, Cisneros GA, Cruzeiro VWD, Darden TA, Duke RE, Giambasu G, Gilson MK, Gohlke H, Goetz AW, Harris R, Izadi S, Izmailov SA, Jin C, Kasavajhala K, Kaymak MC, King E, Koval KP. Amber 2021. Univ California, San Fr. 2021.

**Supplementary Figures**


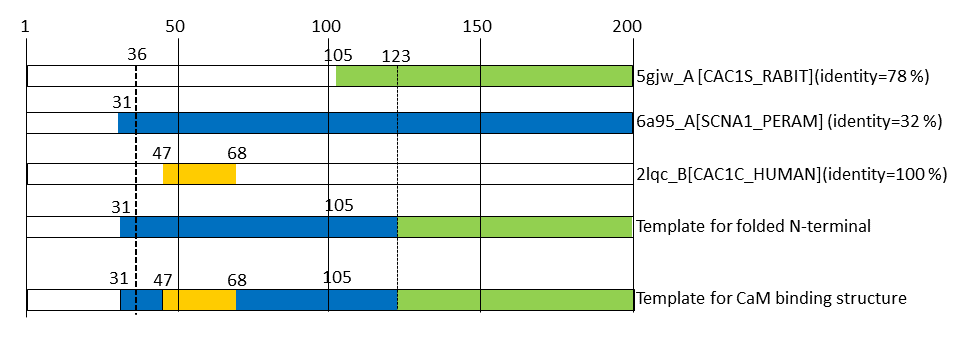


**Fig. S1**. N-terminal region (1-200) of Ca_v_1.2 channel. The amino acid sequence is taken from the RefSeq protein NP_001123312.1. Aligned regions for three homologous 3D structures are shown (5gjw chain A, 6a95 chain A and 2lqc chain B). Chimeric template structures to build the folded N-terminal structure and the CaM binding structure are also shown.


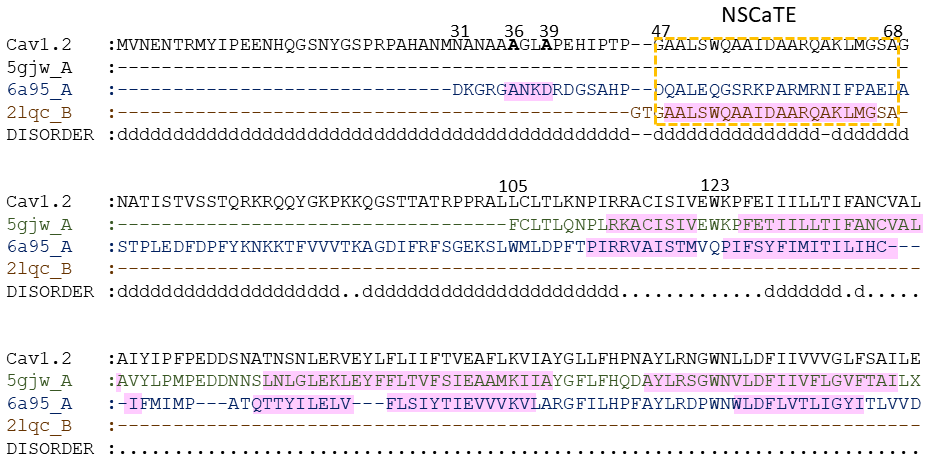


**Fig. S2**. A multiple sequence alignment of N-terminal region of Ca_v_1.2 channel and three homologous 3D structures (5gjw chain A, 6a95 chain A and 2lqc chain B). Alignments are calculated by PSI-BLAST. Predicted intrinsically disordered region were shown as ‘d’ at the bottom line, obtained by DISOPRED 3.16 [6]. α−helix regions are marked in pink.


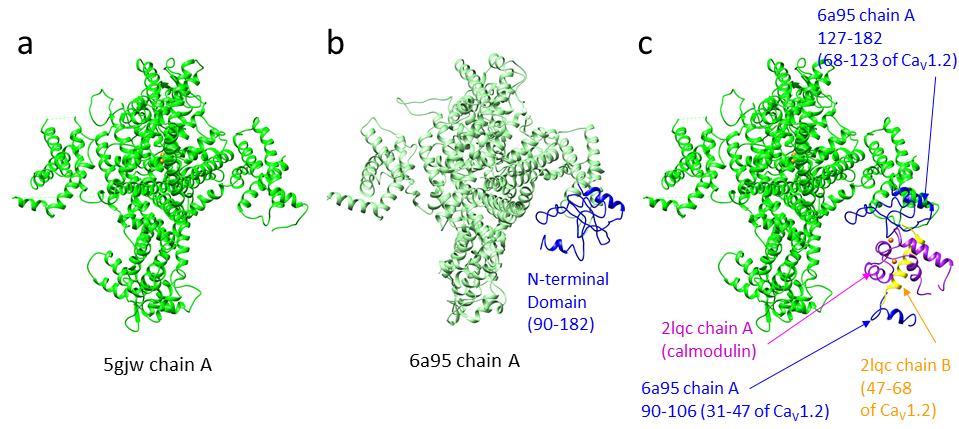


**Fig. S3.** Homologous template 3D structures used for homology modeling of Ca_V_1.2 channel. **a**. rabbit Ca_V_1.1 channel (PDB ID:5gjw). **b**. cockroach Na_v_ channel (PDB ID:6a95) **c**. a template for the CaM-binding structure, which is composed of 90-106 region of 6a95 chain A, NSCaTE region of 2lqc chain B (yellow), 127-182 region of 6a95 chain A (blue), and 5gjw (green). The structure of calmodulin of 2lqc chain A is also shown (magenta).

**
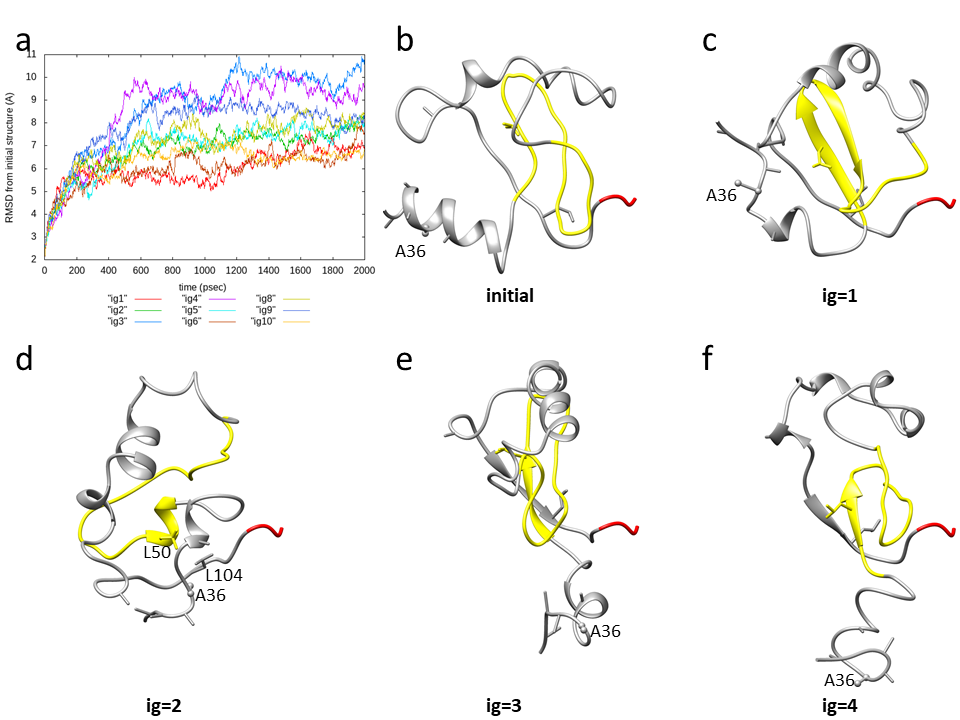
**

**
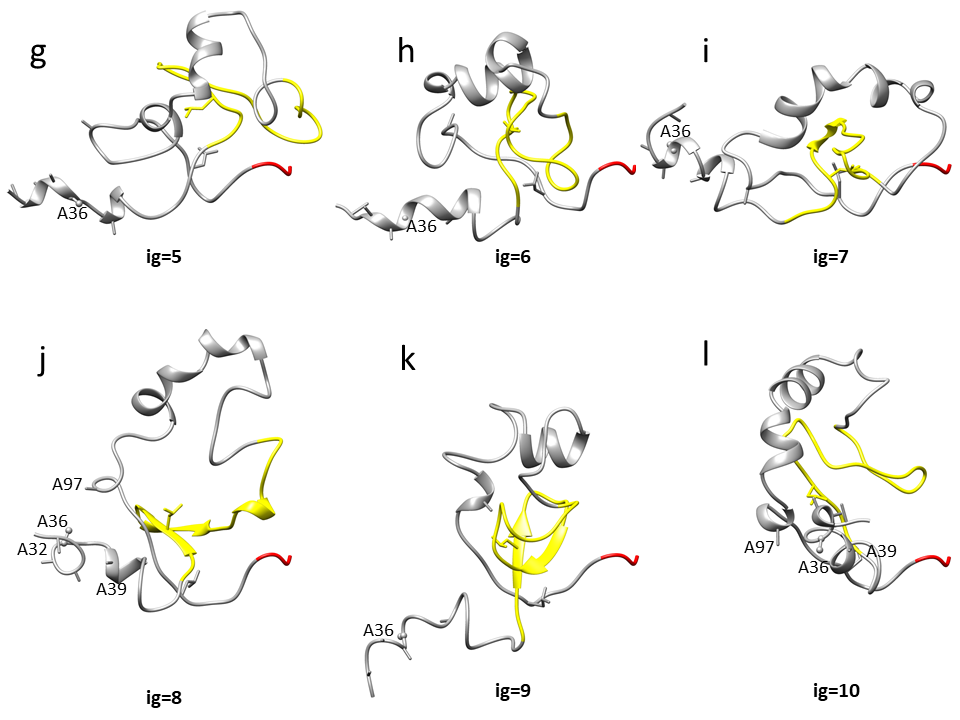
 Fig. S4.** Molecular dynamics (MD) simulations of 31-111 region of Ca_v_1.2 channel. **a**. RMSD values of heavy atoms from the initial structure. 300 K equilibrating simulations were performed for 2 nsec with positional restraints of the three residues (99-101) of the C-terminal with different seed numbers for pseudo-random number generator (ig=1, 2, …, 10). **b**. the template-based 3D model used for the initial structure of MD. It was built using 90-182 region of PDB ID 6a97 chain A. NSCaTE region (47-68) is colored in yellow. The red three residues (109-111) had positional restraints during the simulation. Solvent accessible surface area of the A36 residue is 57 Å^2^. Final structures are shown for the seed number ig=1 (**c**), ig=2 (**d**), ig=3 (**e**), ig=4 (**f**), ig=5 (**g**), ig=6 (**h**), ig=7 (**i**), ig=8 (**j**), ig=9 (**k**), and ig=10 (**l**). In the structure of ig=2(**d**), the A36 has hydrophobic contacts with L50 and L104. In the structures of ig=8 (**j**) and ig=10 (**l**), A36 has a hydrophobic contact with A97. Solvent accessible surface areas of the A36 residue are 38 Å^2^ for ig=2(**d**), 62 Å^2^ for ig=8(**j**), and 50 Å^2^ for ig=10(**l**).
